# Structure–function analyses reveal that a glucuronoyl esterase from *Teredinibacter turnerae* interacts with carbohydrates and aromatic compounds

**DOI:** 10.1074/jbc.RA119.007831

**Published:** 2019-02-27

**Authors:** Jenny Arnling Bååth, Scott Mazurkewich, Jens-Christian Navarro Poulsen, Lisbeth Olsson, Leila Lo Leggio, Johan Larsbrink

**Affiliations:** From the ‡ Wallenberg Wood Science Center, Division of Industrial Biotechnology, Department of Biology and Biological Engineering, Chalmers University of Technology, SE-412 96 Gothenburg, Sweden and; § Department of Chemistry, University of Copenhagen, DK-2100 Copenhagen, Denmark

**Keywords:** protein structure, enzyme kinetics, carbohydrate, plant cell wall, enzyme structure, enzyme mechanism, enzyme mutation, biotechnology, carbohydrate esterase, carbohydrate-active enzymes, CE15, glucuronoyl esterase, lignin-carbohydrate complexes, uronic acid

## Abstract

Glucuronoyl esterases (GEs) catalyze the cleavage of ester linkages found between lignin and glucuronic acid moieties on glucuronoxylan in plant biomass. As such, GEs represent promising biochemical tools in industrial processing of these recalcitrant resources. However, details on how GEs interact with their natural substrates are sparse, calling for thorough structure-function studies. Presented here is the structure and biochemical characterization of a GE, *Tt*CE15A, from the bacterium *Teredinibacter turnerae*, a symbiont of wood-boring shipworms. To gain deeper insight into enzyme–substrate interactions, inhibition studies were performed with both the WT *Tt*CE15A and variants in which we, by using site-directed mutagenesis, substituted residues suggested to have key roles in binding to or interacting with the aromatic and carbohydrate structures of its uronic acid ester substrates. Our results support the hypothesis that two aromatic residues (Phe-174 and Trp-376), conserved in bacterial GEs, interact with aromatic and carbohydrate structures of these substrates in the enzyme active site, respectively. The solved crystal structure of *Tt*CE15A revealed features previously not observed in either fungal or bacterial GEs, with a large inserted N-terminal region neighboring the active site and a differently positioned residue of the catalytic triad. The findings highlight key interactions between GEs and complex lignin-carbohydrate ester substrates and advance our understanding of the substrate specificities of these enzymes in biomass conversion.

## Introduction

**Figure 1.**
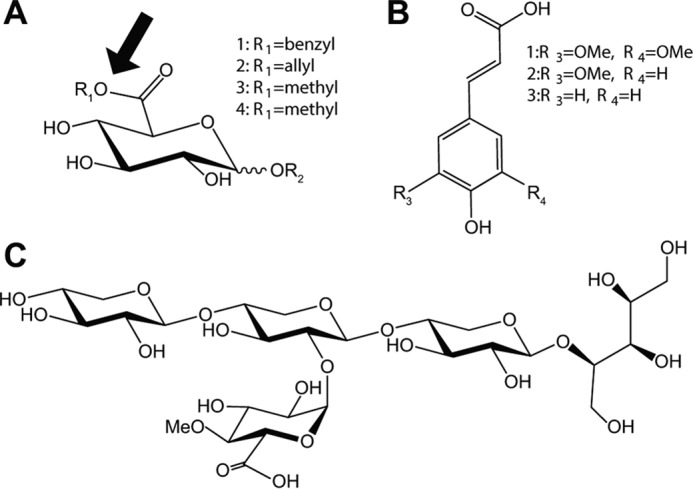
**Substrates used for assaying GE activity and compounds used as inhibitors for GE reactions.** Esters of uronic acids used for assaying enzyme activity are shown: *1*, BnzGlcA; *2*, AllylGlcA; *3*, MeGlcA; *4*, MeGalA (*A*). The position of the OH group linked to the fourth carbon is equatorial for esters of GlcA and axial for GalA. The fourth position of GlcA is often methylated in native lignocellulose. *R_2_* represents H in the model substrates but indicates the position where 4-*O*-MeGlcA is α-1,2–linked to the xylan backbone. The site of GE attack is indicated with an *arrow*. Hydroxycinnamic acids used as GE inhibitors are shown: *1*, SA; *2*, FA; *3*, *p*CoA (*B*) and XUXX_R_ (*C*). *B* and *C* compounds were added in increasing concentrations to enzymatic assays with BnzGlcA to investigate any inhibitory effect on GE activity with aromatic and carbohydrate compounds.

Glucuronoyl esterases (GEs)^3^ are enzymes that act on esters of d-glucuronic acid (GlcA) (1) and belong to the carbohydrate esterase family 15 (CE15) in the carbohydrate-active enzymes database (CAZy; www.cazy.org^4^ (2)). They have been shown to cleave lignin-carbohydrate (LC) ester bonds between 4-*O*-methyl-glucuronoyl moieties in xylan and alcohol moieties in lignin (see Fig. 1*A*) and are therefore suggested as promising tools for reducing plant biomass recalcitrance in industrial settings (3–5). To date, 27 GEs of both fungal and bacterial origin have been biochemically characterized on ester-linked GlcA substrates with varying levels of substitutions and complexity (6–20).

Since the discovery of GEs over a decade ago, the majority of research has focused on fungal GEs, whereas the diversity of bacterial CE15 enzymes has only recently begun to be explored (17, 20, 21). We recently published a comprehensive study on bacterial GEs where 10 novel CE15 enzymes with as low as 25% sequence identity among them were systematically characterized on esters of uronic acids (20). The bacterial GEs across the CE15 family demonstrated similarities and differences in substrate specificity, correlating with their phylogenetic positioning. The study also produced two novel CE15 protein structures (*Ot*CE15A from *Opitutus terrae* and *Su*CE15C from *Solibacter usitatus*), which together with the recent structural determination of MZ0003, a CE15 member from a marine arctic metagenome study (21), represent the only bacterial CE15 structures available thus far.

Three factors have been argued as important for GE substrate specificity regarding substitutions on the GlcA moiety: the length of the carbohydrate portion linked to the anomeric carbon (8, 16, 22), the properties of the alcohol (lignin-derived) portion of the ester, and the presence of a 4-*O*-methyl substituent on the glucuronoate moiety (see Fig. 1*A*). Although studies indicate that GEs have minimal specificity toward the presence or type of carbohydrates linked to the anomeric position of the GlcA, the enzymes tend to have increased specificity toward bulkier alcohol moieties and a methylation on the 4-OH position (8–10, 13, 14, 22). However, recent characterizations have revealed several bacterial CE15 enzymes with high activities on ester substrates that lack 4-*O*-methylation, indicating a degree of diversity across the family (20). For fungal GEs, the configuration of the C4 hydroxyl moiety has been suggested to be important due to the lack of activity on esters of d-galacturonic acid (GalA) (6, 8). Similar preferences have been reported for some bacterial GEs, although several bacterial CE15 enzymes are promiscuous and exhibit equal activities on both GlcA- and GalA-derived substrates (20). Additionally, two bacterial CE15 enzymes have significant acetyl esterase activity in addition to their GE activity, which again indicates a broader substrate specificity among bacterial CE15 enzymes (17, 23). The differences in substrate specificity among CE15 enzymes potentially point toward different biological roles, especially in cases where multiple CE15 enzymes are encoded by a single microorganism.

Five GE structures have been determined by X-ray crystallography (20, 21, 24, 25). All of them share a typical α/β-hydrolase fold, with a solvent-exposed active site containing the catalytic residues. The three bacterial structures differ from the two fungal structures (*Tr*GE (Cip2) from *Trichoderma reesei* and *St*GE2 from *Thermothelomyces thermophila* (previously *Myceliophthora thermophila* and *Sporotrichum thermophile*)) by three inserted regions and key active site differences. Of the three regions, a prominent insertion of ∼45 residues creates a ridge on one side of the active site, resulting in a significantly deeper cleft compared with the fungal structures (20, 21). The active sites of the bacterial structures have further been proposed to be more flexible due to the absence of disulfide bridges that are present in the fungal structures (21). *Tr*GE (Cip2), *St*GE2, and the bacterial *Ot*CE15A and *Su*CE15C all share the same catalytic triad: serine-histidine-glutamate. The triad is, however, not fully conserved in all CE15 enzymes, as glutamate has been found to be replaced by other amino acids in a number of enzymes. In MZ0003, a cysteine is found at the equivalent position of the canonical glutamate, and a closely positioned aspartate is suggested to act as the acidic residue in the reaction (21). Interestingly, an aspartate is found at the equivalent noncanonical position in *Ot*CE15A and *Su*CE15C in addition to the canonical glutamate.

Only a fraction of the CE15 members listed in CAZy have been characterized, and the properties of different GEs are currently difficult to compare due to use of various GlcA ester model substrates and different biochemical analyses and experimental conditions. In addition, a lack of detailed structure-function studies precludes understanding of the molecular interactions between these enzymes and their substrates, which is essential for understanding their potential role(s) in microbial biomass conversion.

The aim of the present study was to investigate structure-function relationships of *Tt*CE15A, a novel CE15 enzyme from the Gram-negative bacterium *Teredinibacter turnerae. T. turnerae* has been isolated from the gills of a wood-boring shipworm, a mollusk that digests lignocellulose in marine environments (26). The bacterium encodes multiple CE15 enzymes (27), which were hypothesized to be novel GEs involved in the degradation of lignocellulose on the sea floor. In the present work, *Tt*CE15A was biochemically characterized on uronic acid ester substrates, and its structure was solved by X-ray crystallography. Residues proposed to interact with both the xylan and lignin portions of natural substrates were investigated through comparative inhibition studies using both the WT enzyme and enzyme variants.

## Results

### Sequence analysis and enzyme production

*T. turnerae* encodes three CE15 enzymes (*Tt*CE15A, -B, and -C; locus tags TERTU_0517, TERTU_3447, and TERTU_3514, respectively). The enzymes are relatively distant in terms of sequence (37% identity between A and B, 25% identity between A and C, and 51% identity between B and C). *Tt*CE15A is phylogenetically more distant to the fungal CE15 enzymes than are the other two *Tt*CE15 enzymes and the previously characterized bacterial members (20). The most similar characterized CE15 enzymes are the bacterial *Ot*CE15D, *Su*CE15B, and *Sl*CE15B and -C from *Spirosoma linguale*, with sequence identities between 39 and 43%. The sequence identities between *Tt*CE15A and the five previously structurally characterized CE15 enzymes range between 30 and 34% (Table S1). *Tt*CE15B and *Tt*CE15C could not be expressed solubly in our experiments, whereas *Tt*CE15A production yielded ∼80 mg of protein/liter of culture broth after nickel-affinity purification.

### Biochemical characterization and substrate specificity of TtCE15A

Kinetic parameters were determined on esters of glucuronic and galacturonic acids as well as 4-nitrophenyl acetate (*p*NP-Ac) (Table 1 and Fig. 1*A*). Benzyl glucuronoate (BnzGlcA) was used to determine the pH optimum, which was shown to be alkaline (pH 8.5; Fig. S1). *Tt*CE15A exhibited a remarkably high substrate turnover number on BnzGlcA, *k*_cat_ = 132 s^−1^, which is more than 2-fold higher than previously reported (58 s^−1^ for *Su*CE15C (20)) on this substrate. The *K_m_* value for BnzGlcA of 3.5 mm was similar to several other characterized bacterial and fungal GEs (14, 20, 28). The substrate affinity decreased drastically for glucuronoate esters with smaller alcohol portions (allyl and methyl), with a *K_m_* of 50 mm for allyl glucuronoate (AllylGlcA) and an unsaturable reaction for methyl glucuronoate (MeGlcA; up to 50 mm), where the *K_m_* value consequently could not be determined. The kinetic data emphasize the apparent importance of a larger and bulkier alcohol part of the ester to reach full activity for *Tt*CE15A. Although the *k*_cat_/*K_m_* was found to be high (between 1 and 40 s^−1^ mm^−1^) for *Tt*CE15A on all three GlcA esters, it was 2–3 orders of magnitude lower using methyl galacturonoate (MeGalA) as a substrate (0.012 s^−1^ mm^−1^; Table 1). This demonstrates that *Tt*CE15A is a true GE, with minimal promiscuous activity for GalA relative to GlcA. *Tt*CE15A was additionally assayed on *p*NP-Ac; however, no significant acetyl esterase activity could be detected.

**Table 1:** **Kinetic parameters of TtCE15A WT and variants on model substrates** Esterase activity was assayed with benzyl, allyl, and methyl esters of GlcA and GalA and *p*NP-Ac (acetyl esterase activity).

Substrate	*Tt*CE15A	*K_m_*	*k*_cat_	*k*_cat_/*K_m_*
		*mm*	*s*^−*1*^	*s*^−*1*^ *m*^−*1*^
BnzGlcA	WT	3.47 ± 0.28	132 ± 3.6	(3.83 ± 0.16) × 10^4^
	S281A	4.00 ± 0.30	0.360 ± 0.010	(9.05 ± 0.06) × 10^1^
	F174A	25.8 ± 1.4	136 ± 4.8	(5.25 ± 0.35) × 10^3^
	F174D	53.1 ± 3.9	43.1 ± 2.4	(8.11 ± 0.75) × 10^2^
	W376A	22.7 ± 3.8	18.3 ± 1.9	(8.03 ± 1.58) × 10^2^
	W376D	15.4 ± 4.0	12.8 ± 1.8	(8.32 ± 2.44) × 10^2^
	E374A	21.8 ± 2.1	4.12 ± 0.25	(1.89 ± 0.22) × 10^2^
	S304E/E374A	12.7 ± 2.0	2.83 ± 0.23	(2.23 ± 0.20) × 10^2^
	S304E	29.4 ± 9.6	1.92 ± 0.42	(6.54 ± 2.57) × 10^1^
AllylGlcA	WT	49.0 ± 1.7	200 ± 4.3	(4.08 ± 0.17) × 10^3^
	F174A	Cannot be saturated up to 50 mm		(4.20 ± 0.02) × 10^2^
	F174D	Cannot be saturated up to 50 mm		(1.22 ± 0.02) × 10^2^
MeGlcA	WT	Cannot be saturated up to 50 mm		(1.03 ± 0.01) × 10^3^
	F174A	Cannot be saturated up to 50 mm		(7.82 ± 0.15) × 10^1^
	F174D	Cannot be saturated up to 50 mm		(3.17 ± 0.05) × 10^1^
MeGalA	WT	Cannot be saturated up to 50 mm		(1.20 ± 0.09) × 10^1^
*p*NP-Ac	WT	Cannot be saturated up to 10 mm		2.50 ± 0.40

### Structural determination

#### 

##### Overall structure

To gain deeper insight into the structure-function relationship of *Tt*CE15A and the CE15 family as a whole, structural characterization was pursued. The structure of a selenomethionine-substituted *Tt*CE15A was determined by X-ray crystallography using SAD phasing (Protein Data Bank (PDB) code 6hsw). *Tt*CE15A has an α/β-hydrolase fold similar to previously determined CE15 structures, consisting of a three-layer sandwich with a solvent-exposed cleft comprising the active site with its catalytic triad. Three *Tt*CE15A molecules were found in the asymmetric unit, with a Cα root mean square deviation below 0.2 Å (all atoms <1 Å), indicating a high degree of similarity between the protein chains.

**Figure 2.**
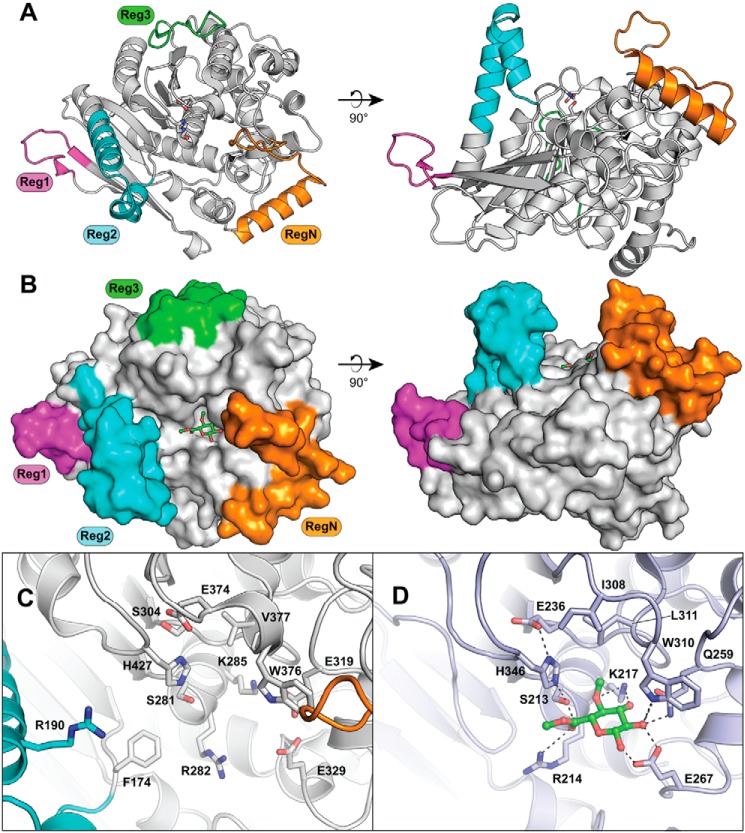
**Structure of *Tt*CE15A.** The overall structure (*A*) and space-filling representation (*B*) with the methyl ester of 4-*O*-methyl glucuronoate substrate shown as *green sticks* in (*B*) were generated from structural alignment with the substrate complex structure of *St*GE2 (PDB code 4g4j). The inserted regions relative to the fungal CE15 enzymes, regions N, 1, 2, and 3, are colored *orange*, *magenta*, *cyan*, and *green*, respectively. Comparison of the active site organization of *Tt*CE15A (*C*) and *St*GE2 (*D*) shows conservation of residues between the enzymes for binding of the glucuronoate moiety, whereas *Tt*CE15A has portions of RegN and Reg2 additionally protruding into the active site.

Like the previously determined bacterial structures (20, 21), *Tt*CE15A has three inserted regions relative to the fungal CE15 structures (Reg1, residues 122–134; Reg2, 175–200; Reg3, 399–415) (Fig. 2, *A* and *B*). Reg1 and Reg3 have structures similar to the corresponding regions in MZ0003, *Ot*CE15A, and *Su*CE15C, whereas Reg2 lacks both sequence and structural similarity with the others (Figs. S2 and S3). Although being shorter in sequence (26 *versus* 40–45 residues), Reg2 of *Tt*CE15A forms a much longer helix compared with the Regs2 of *Ot*CE15A, *Su*CE15C, and MZ0003. The longer Reg2 helix present in *Tt*CE15A builds up a larger ridge around the previously proposed lignin-binding region and thus presents a deeper substrate-binding groove compared with previously solved structures (Fig. 2, *A* and *B*, and Fig. S2). On the ridge built up by Reg2, multiple hydrophobic residues are found on the side facing the active site (Phe-174, Ile-178, Trp-179, Phe-182, Met-186, and Ile-194), whereas the opposite face is more hydrophilic. At the N terminus, *Tt*CE15A has an additional extension (RegN, residues 27–55) consisting of ∼30 residues neighboring the previously proposed xylan-binding region (20). RegN forms a further deepening of the active site pocket opposite Reg2, a feature not present in any previously determined CE15 structure (Fig. 2, *A* and *B*). RegN consists of several hydrophilic residues, which may be involved in binding of xylan or xylooligosaccharides.

##### Active site pocket

Although all CE15 members exhibit variability in the hydrophilic residues surrounding the active site, several key residues observed to interact with the methyl esterified 4-*O*-methyl glucuronoate ligand in the structurally solved complex with *St*GE2 (25) are conserved in *Tt*CE15A: Arg-282, interacting with the glucuronoyl carboxylate moiety; Lys-285, hydrogen bonding to both the 3-OH and the oxygen of the 4-*O*-methyl group; and Trp-376, hydrogen bonding to the glucuronoyl 2-OH (Fig. 2, *C* and *D*). These residues are conserved across all structurally characterized CE15 members (Fig. S3). The conserved tryptophan has also been suggested to interact with the xylan portion of the substrate (20). The recent structural determinations of the first bacterial CE15 enzymes identified a phenylalanine close to the catalytic histidine and serine within the pocket of Reg2 that has been proposed to interact with aromatic components of the LC substrates (20, 21). Although absent in fungal CE15 enzymes, the residue is conserved among all bacterial CE15 members characterized to date, including *Tt*CE15A (Phe-174; Fig. S3).

**Figure 3.**
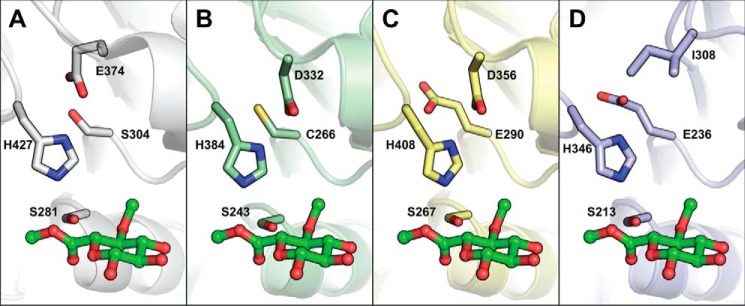
**Comparison of catalytic residues among selected structurally characterized CE15 members.** The *Tt*CE15A (*A*), MZ0003 (*B*; PDB code 6ehn), *Ot*CE15A (*C*; PDB code 6gs0), and *St*GE2 (*D*; PDB code 4g4g) are shown in *gray*, *green*, *yellow*, and *blue*, respectively. The enzymes are shown relative to the methyl ester of 4-*O*-methyl glucuronoate substrate shown as *green sticks*, generated from structural alignment with the complex structure of *St*GE2 (PDB code 4g4j). The catalytic residues and equivalently placed residues are shown in *sticks*. Notably, the canonical acidic residue observed in *St*GE2 is absent in *Tt*CE15A and MZ0003, and instead the acidic residue is found on a different loop, and in *Ot*CE15A, acidic residues are found in both positions.

The serine and histidine residues of the catalytic triad found in all previously solved structures (fungal and bacterial) are conserved in *Tt*CE15A (Ser-281 and His-427; Fig. 3). However, the proposed canonical glutamate of the catalytic triad is absent (similar to MZ0003), and this position is occupied by a serine residue (Ser-304) (Fig. 3*A*). In MZ0003, a closely positioned aspartate (Asp-332) has been proposed to act as the acidic residue of the catalytic triad in place of the missing canonical glutamate (confirmed by mutagenesis) (Fig. 3*B*). In *Tt*CE15A, a glutamate, Glu-374, is found in an equivalent position, which would likely fulfill the same role. Interestingly, *Ot*CE15A and *Su*CE15C both have an aspartate at the equivalent position of the Glu-374 of *Tt*CE15A and the Asp-332 of MZ0003 in addition to having the glutamate of the canonical catalytic triad (Fig. 3*C*). The fungal *St*GE2 and Cip2 structures both have isoleucine residues in the equivalent position of the noncanonical glutamate/aspartate (Fig. 3*D*).

### Investigation of the noncanonical catalytic triad acidic residue

Due to the absence of a canonical glutamate in *Tt*CE15A and the positioning of Glu-374 in the active site, the latter residue was investigated as a potential noncanonical acidic residue in the catalytic triad. Consistent with the proposed role of the residue in the catalytic triad, an E374A variant had a 100-fold decrease in *k*_cat_ (4.1 s^−1^) together with an increased *K_m_* (21.8 mm) for the model substrate BnzGlcA (Table 1). Similar to previous reports for other serine esterases, substituting the acidic residue may strongly reduce, but not eliminate, the activity of the enzyme as other residues or water molecules can partially substitute for the missing functional group (29). An S304E/E374A variant was produced to assess whether the native high turnover number could be restored by introduction of a glutamate at the position of the canonical acidic residue. However, the turnover number of the S304E/E374A variant on BnzGlcA was not recovered (*k*_cat_ = 2.8 s^−1^; *K_m_* = 12.7 mm). Furthermore, a variant harboring solely the S304E substitution, *i.e.* possessing a glutamate in both the canonical and noncanonical positions (Glu-304 and Glu-374) was additionally catalytically crippled (*k*_cat_ = 1.9 s^−1^; *K_m_* = 29.4 mm) (Table 1). Collectively, the results indicate that the *Tt*CE15A is fine-tuned to utilize Glu-374 as the acidic residue in the catalytic mechanism, supporting the enzyme's high turnover rate, and that the utilization of the residue in this position is distinct from CE15 members exhibiting the canonical acidic residue.

### Interaction of Trp-376 with the glucuronoate moiety

In the fungal *St*GE2, the conserved tryptophan in the active site (Trp-376 in *Tt*CE15A) aids in binding GlcA through hydrogen bonding between the indole nitrogen and the 2-OH of the sugar (25). Enzyme variants of the *Tt*CE15A tryptophan (W376A and W376D) were catalytically compromised, displaying 5-fold increased *K_m_* values and 10-fold decreased *k*_cat_ values (Table 1). These results indicate that the tryptophan makes a considerable contribution to the interaction with GlcA, which when perturbed reduces catalytic efficiency on the model substrates.

### Investigation of the putative lignin-binding pocket

Because the bulky benzyl moiety on the model substrate BnzGlcA appears to be crucial for substrate affinity, the possible inhibition of the action of *Tt*CE15A on BnzGlcA by aromatic molecules was investigated. Benzyl alcohol and three different hydroxycinnamic acids, ferulic (FA), sinapic (SA), and *p*-coumaric acids (*p*CoA) (Fig. 1*B*), common phenolic compounds in lignocellulose-derived streams, were investigated as potential inhibitors of the BnzGlcA esterase reaction. We determined the IC_50_ values (amount of inhibitor required to reduce the reaction rate by 50%) for these reactions, where possible, to be able to estimate the extent of the inhibitory effect(s).

**Figure 4.**
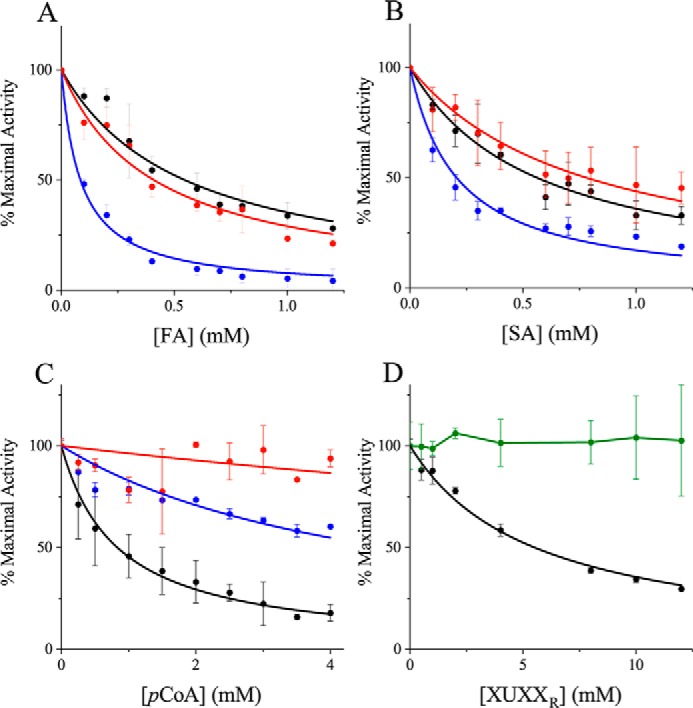
**Inhibition of *Tt*CE15A with aromatic compounds and xylooligosaccharides.** Inhibition of the WT enzyme activity (*black*) compared with F174A (*blue*) and F174D (*red*) by FA (*A*), SA (*B*), and *p*CoA (*C*) and inhibition of *Tt*CE15A WT activity (*black*) compared with W376A (*green*) by XUXX_R_ (*D*) are shown. The inhibitors were added in increasing concentrations to 0.4 mm BnzGlcA, up to 1.2 mm FA or SA, 4 mm *p*CoA, and 12 mm XUXX_R_. The inhibitory effect of the compounds was calculated by nonlinear regression, fitting Equation 1 (see “Experimental procedures”) to the data (not possible for W376A with XUXX_R_). *Error bars* represent S.D. from the mean value of duplicate measurements. The data are normalized to facilitate comparison, where 100% maximal activity corresponds to the following rates (*v*/[*E*]*_t_*): 11.1 (WT), 1.7 (F174A), 0.3 (F174D), and 3.0 s^−1^ (W376A). Similar or even stronger inhibition by FA and SA was observed for the Phe-174 variants compared with the WT enzyme. However, the variants lacking Phe-174 or Trp-376 were less inhibited by *p*CoA and XUXX_R_ than the WT enzyme.

Inhibition was observed when FA, SA, and *p*CoA were added to the reactions individually. The IC_50_ values for these compounds were 0.55, 0.56, and 0.83 mm, respectively, in reactions containing 0.4 mm BnzGlcA (Table 2 and Fig. 4, *A–C*). No inhibition of *Tt*CE15A activity was observed when adding benzyl alcohol (the product of the BnzGlcA reaction; up to 12 mm). The lower IC_50_ values of FA and SA as inhibitors compared with *p*CoA suggests that the methoxy groups of FA and SA may provide interactions with the active site residues. The overall results from the inhibition experiments using hydroxycinnamic acids indicated that there are interactions between the active site of *Tt*CE15A and aromatic molecules. Following previous docking studies performed on *Ot*CE15A using a benzyl esterified glucuronoxylotriose oligosaccharide (20), specific interactions of a phenylalanine residue with the aromatic portions of LC structures were postulated. Enzyme variants of the equivalently positioned Phe-174 of *Tt*CE15A were therefore created to investigate interaction with the benzyl moiety.

**Table 2:** **IC_50_ values of different compounds on the TtCE15A WT enzyme and enzyme variants using BnzGlcA as substrate** NM, not measured; NI, no/minor inhibition detected (>87% retained enzyme activity after maximum addition of inhibitor).

*Tt*CE15A	FA	SA	*p*CoA	XUXX_R_
	*mm*	*mm*	*mm*	*mm*
WT	0.55 ± 0.045	0.56 ± 0.030	0.83 ± 0.04	5.51 ± 0.28
F174A	0.084 ± 0.005	0.19 ± 0.022	4.84 ± 0.47	NM
F174D	0.40 ± 0.040	0.77 ± 0.064	26.0 ± 12.0	NM
W376A	NM	NM	NM	NI

Substitution of Phe-174 with alanine (F174A) resulted in a 10-fold higher *K_m_* (26 mm) for BnzGlcA, whereas the *k*_cat_ value remained similar (136 s^−1^; Table 1). A second variant, F174D, was even more catalytically impaired, with a 15-fold increase in *K_m_* (53 mm) and a halved *k*_cat_ value (43 s^−1^). Both variants exhibited lower *k*_cat_/*K_m_* values by an order of magnitude or more compared with the WT enzyme assayed using AllylGlcA or MeGlcA as substrates. Differential scanning fluorimetry confirmed similar melting temperatures (*T_m_*) for the Phe-174 variants (52–54 °C) and the WT enzyme (52 °C), suggesting that the reduced catalytic efficiencies are not due to significantly compromised enzyme structures. Collectively, the results suggest that Phe-174 is important not only for binding of aromatic benzyl moieties, but it also affects the catalytic action on smaller substrates.

The inhibition studies using hydroxycinnamic acids were repeated for the two enzyme variants F174A and F174D (Table 2 and Fig. 4, *A–C*), with a hypothesis that the inhibitory effects would be reduced compared with the WT enzyme. Both variants were less inhibited by *p*CoA with higher IC_50_ values observed (6-fold for F174A and 30-fold for F174D; Table 2 and Fig. 4*C*). However, the IC_50_ values for FA and SA were similar (F174D) or significantly lowered (F174A; 6-fold decrease) compared with the reactions with the WT enzyme (Table 2 and Fig. 4, *A* and *B*). The results are puzzling and do not clearly indicate an involvement of Phe-174 in binding FA or SA. However, it is possible that the increased space resulting from replacing Phe-174 with smaller residues enables the methoxylated FA and SA to bind closer to the catalytic residues and thereby more easily perturb the catalytic action of the enzyme variants. To fully elucidate the role of Phe-174 in binding of aromatic structures, enzyme–ligand structures are required. Despite extensive efforts, we have thus far been unable to obtain ligand complexes with *Tt*CE15A, but future experiments might illuminate which residues provide key contributions in substrate recognition.

### Investigation of potential xylan-interacting residues

We previously postulated that GEs may interact with a larger carbohydrate portion of xylan than just the glucuronoate moiety (20). To examine how *Tt*CE15A may interact with mono- and oligosaccharides, potential inhibition of the hydrolysis of BnzGlcA was assayed using a range of carbohydrates: arabinose, glucose, xylose, xylotriose, and the reduced 2^3^-(4-*O*-methyl-d-glucuronyl)-α-d-xylotetraitol (XUXX_R_; Fig. 1*C*). XUXX_R_ mimics the carbohydrate portion of native LC ester structures. None of the monosaccharides nor xylotriose (up to 12 mm) was able to inhibit the reaction of the enzyme with BnzGlcA (at 0.4 mm), but addition of XUXX_R_ to the reactions reduced the activity of *Tt*CE15A on BnzGlcA significantly (IC_50_ = 5.51 mm; Table 2 and Fig. 4*D*).

Interactions between the conserved active site tryptophan and the carbohydrate portion of LC structures have previously been proposed from our docking simulations on *Ot*CE15A (20). In *Tt*CE15A, to investigate whether the conserved tryptophan (Trp-376) may interact with the xylose moiety α-1,2–linked to 4-*O*-MeGlcA, we performed inhibition experiments with the previously described W376A enzyme variant. In contrast to the WT *Tt*CE15A enzyme, addition of XUXX_R_ did not inhibit the activity of W376A, which retained 100% activity even in the presence of 12 mm XUXX_R_ (Fig. 4*D*). This observation supports the hypothesis that Trp-376 is involved in positioning and/or binding to the main chain xylan or xylooligosaccharides in native cell wall–derived substrates.

We attempted to obtain data for the binding of GlcA and benzyl alcohol, respectively, to *Tt*CE15A using isothermal titration calorimetry and surface plasmon resonance measurements. Unfortunately, neither experimental setup yielded conclusive data. Possibly, this was a result of a relatively weak binding of *Tt*CE15A to these molecules, as indicated by the relatively high *K_m_* value for BnzGlcA (Table 1).

## Discussion

The proposed biological role of GEs is to hydrolyze ester bonds between lignin and glucuronoxylan in plant cell walls, which would greatly reduce the cell wall recalcitrance. However, information on how GEs interact with their native substrates is still lacking, primarily due to difficulties with substrate acquisition and suitable detection methods. The bacterial CE15 enzyme examined in the present work, *Tt*CE15A, is the most dissimilar, in terms of primary structure, to the fungal enzymes characterized to date. Our investigations revealed a remarkably high substrate turnover rate of *Tt*CE15A on the typical model substrate BnzGlcA as well as a strong preference for larger aromatic ester substituents on GlcA. The model substrates used in this study have been used to characterize the majority of previously studied bacterial GEs, which allows for comparisons of the kinetic behavior of *Tt*CE15A with several closely and distantly related characterized CE15 enzymes. As expected, *Tt*CE15A exhibited properties highly similar to those of the two GEs most similar in primary structure, *Ot*CE15D and *Sl*CE15B, by strongly preferring the BnzGlcA substrate over smaller substrates and displaying a strict preference for esters of GlcA (no activity detected for MeGalA) (20).

The crystal structure of *Tt*CE15A revealed features previously not observed in either fungal or bacterial GEs. The lack of a canonical catalytic glutamate residue and the existence of a noncanonically positioned carboxylic acid in a catalytically favorable orientation have previously been observed in the MZ0003 enzyme (21). Here, we demonstrated through mutagenesis that the noncanonical Glu-274 in *Tt*CE15A is the general acid of the catalytic triad. However, attempting to switch the position of the glutamate to the canonical position typically encountered in GEs (S304E/E374A) did not significantly increase the activity above that of the E374A variant, indicating that the position of the acidic residue is not interchangeable. Interestingly, there are aspartate residues present in the equivalent position of Glu-374 in both of the previously solved structures of *Ot*CE15A and *Su*CE15C, but whether these residues (Asp-356 in *Ot*CE15A and Asp-346 in *Su*CE15C) could participate in catalysis is not known. Because the activity is only reduced but not abolished in the absence of the catalytic carboxylate, this has likely allowed a later rescuing by change in a neighboring side chain and the shift of the positioning of the catalytic acid in GEs.

The inhibition studies performed for *Tt*CE15A-catalyzed reactions revealed, for the first time, that small aromatic compounds as well as specific oligosaccharides can inhibit GE activity. The results strengthen the prevailing hypothesis that GEs interact with both aromatic lignin and hydrophilic carbohydrate parts of the substrate, although the exact nature of these interactions remains elusive. We have demonstrated that a tryptophan residue (Trp-376) is important for hydrolysis of small substrates, and the inhibition profiles are consistent with it also being important for binding GlcA-decorated xylotriose. The length of the xylan chain linked to the anomeric position of the GlcA may possibly be of less importance, as has previously been shown by the comparable kinetic values observed for the GE *Sc*GE (from *Schizophyllum commune*) on esters comprising either xylose- or xylooligosaccharide structures (22). The most striking structural feature of *Tt*CE15A was the presence of a large N-terminal extension opposite Reg2. An N-terminal loop of this size is unprecedented among characterized GEs to date. Residues of Reg2 have previously been proposed to interact with lignin or lignin fragments, and the positioning of RegN suggests a role in binding of carbohydrate moieties of LC complexes. Overall, the inserts of *Tt*CE15A lead to a structure with a deep substrate-binding groove and possibly extensive interactions with both lignin and carbohydrates. What biological consequences this deepened active site topology may have, compared with the open-face active sites of structurally solved fungal enzymes, are currently not understood. As a symbiont of marine shipworms, *T. turnerae* is likely presented with partially solubilized wood-derived material, which may be rich also in LC fragments. Possibly, *Tt*CE15A has evolved to target smaller LC fragments in solution that can be captured by the clamplike active site, whereas the studied fungal enzymes would be more adapted to act on larger, solid substrates that are more abundant in terrestrial environments.

The two additional CE15 enzymes encoded by *T. turnerae*, *Tt*CE15B and *Tt*CE15C, might complement *Tt*CE15A in the biology of the bacterium. A multiple sequence alignment (Fig. S4) shows some noteworthy differences among the three enzymes. *Tt*CE15B and *Tt*CE15C possess, unlike *Tt*CE15A, the canonically positioned glutamate, and *Tt*CE15C additionally has an aspartate in the noncanonical position, similar to the active sites of *Ot*CE15A and *Su*CE15C. Notably, the conserved tryptophan suggested to bind xylan chains in previously characterized GEs is not conserved in either *Tt*CE15B or *Tt*CE15C, and from the multiple sequence alignment, it appears that both *Tt*CE15B and *Tt*CE15C lack the putative lignin-binding phenylalanine residue conserved among characterized bacterial CE15 members. Moreover, the lack of inserted regions and the presence of possible disulfide bridges in both *Tt*CE15B and *Tt*CE15C are features shared with characterized fungal GEs. Previous investigations of *S. linguale*, a species also encoding three CE15 genes, showed that expression of each gene depends on the type of carbon source to which the host is exposed (20). Taken together, it is reasonable to assume that the different *T. turnerae* CE15 genes are nonredundant and exhibit unique substrate specificities, although further investigations are required to elucidate the roles of these enzymes in biomass decomposition.

In this work, we have provided a detailed structure–function study of a novel and highly active GE from CE15, with a proposed role in separation of carbohydrates from lignin. From structural analysis and biochemical characterization followed by site-directed mutagenesis of key active site residues, new insights into both the structural diversity and key residues involved in substrate binding in GEs were attained. In future studies, comparisons of active site residues of various GE enzymes could yield a more comprehensive understanding of the general interactions between GEs and their complex substrates consisting of both hydrophilic carbohydrates and hydrophobic lignin, as would synthesis of more varied model substrates with higher similarity to the proposed native LC structures. Structures of GEs in complex with biologically relevant ligands would further illuminate our understanding of how GEs interact with their natural substrates.

## Experimental procedures

### Cloning, expression, and purification of TtCE15A

The *Tt*CE15A gene was amplified from genomic DNA of *T. turnerae* DSM 15142 (DSMZ) by PCR (primers are listed in Table S2). The product was cloned into a modified pET-28a vector (In-Fusion HD kit, Clontech), containing N-terminal His_6_ tags and tobacco etch virus protease cleavage sites, and gene expression was performed in *Escherichia coli* BL21(λDE3). Cells were grown in lysogeny broth (LB), at 37 °C with 200 rpm shaking, containing 50 μg/ml kanamycin. At *A*_600_ of ∼0.5, expression was induced by the addition of isopropyl β-d-1-thiogalactopyranoside (0.2 mm final concentration), and the cells were incubated at 16 °C overnight. Cells were harvested by centrifugation (5000 × *g*, 10 min); resuspended in 20 mm Tris buffer (pH 8) containing 250 mm NaCl, 5 μg/ml lysozyme, and 10 μg/ml DNase; and disrupted by sonication. Cell debris was removed by centrifugation (18,000 × *g*, 15 min), and proteins were purified using immobilized metal ion affinity chromatography on an ÄKTA system (GE Healthcare) using 5-ml HisTrap^TM^ Excel columns. 50 mm Tris (pH 8), 250 mm NaCl was used as binding buffer, and elution was performed in one step with 250 mm imidazole. The purified fraction was dialyzed into 20 mm Tris buffer (pH 8).

### Site-directed mutagenesis

The plasmid containing *Tt*CE15A was used as template to create *Tt*CE15A variants according to the QuikChange II site-directed mutagenesis method (Agilent Technologies) using primers listed in Table S2. Mutant strand synthesis reaction (thermal cycling) with Phusion high-fidelity DNA polymerase (Thermo Fisher) was followed by DpnI digestion (Thermo Fisher) of the amplification products and transformation into Stellar^TM^ competent cells (Clontech). Gene expression was performed in *E. coli* BL21(λDE3), and the protein was purified as described for the WT enzyme. Differential scanning fluorimetry with SYPRO^TM^ Orange protein stain (Invitrogen) was performed to monitor the thermal transition of the *Tt*CE15A enzyme variants. Measurements were performed in triplicates on a Stratagene MX3005P qPCR instrument (Agilent Technologies) with gradual heating of the sample plate (1 °C/min) from 25 to 90 °C.

### Enzyme assays

Glucuronoyl esterase activity was assayed with the substrates BnzGlcA, AllylGlcA, MeGlcA, and MeGalA (Carbosynth) and monitored continuously with the K-URONIC kit (Megazyme) as reported previously (20). Although the pH optimum of *Tt*CE15A was at pH 8.5, standard assays were kept at pH 7.5 due to instability of the ester substrates at the higher pH. The substrates were dissolved in 100% DMSO, and all reactions contained less than 10% DMSO. pH dependence profiles were generated with 2 mm BnzGlcA in a three-component buffer containing 25 mm acetic acid, 25 mm MES, and 50 mm Tris-HCl (pH 4.5–9.5) (30). Acetyl esterase activity was assayed using *p*NP-Ac (Sigma-Aldrich) as described previously (20). Curve fitting and kinetic parameters were calculated using OriginPro 2017 software (OriginLab).

### Crystallization and data collection

*Tt*CE15A was screened for crystallization in MRC two-drop crystallization plates (Molecular Dimensions) using an Oryx 8 robot (Douglas Instruments). Sitting drops (0.3 μl) were mixed with protein:reservoir volume ratios of 3:1 or 1:1 using 35 mg/ml *Tt*CE15A in 20 mm Tris (pH 8.0). Hits from Morpheus screens (Molecular Dimensions) were optimized, and final crystallization conditions were as follows: 0.09 m halogens, 0.1 mbuffer system 1 (imidazole and MES), and 50% (v/v) precipitant mixture 2 (40% (v/v) ethylene glycol and 20% (w/v) PEG 8000) (31). Crystals were mounted using nylon loops and flash frozen in liquid N_2_ in the absence of additional cryoprotectant. An initial data set, which diffracted to ≈2.5 Å, was collected on ID30B at the European Synchrotron Radiation Facility (ESRF), Grenoble, France. Molecular replacement using the previously determined fungal and bacterial CE15 structures as templates (25–45% identity) was unsuccessful, and collection of anomalous data was pursued. The pET-28a construct was transformed into *E. coli* T7 Express Crystal (methionine auxotroph; New England Biolabs), and the protein was expressed in minimal medium containing seleno-l-methionine (SeMet) according to the supplier's recommendations (Molecular Dimensions). *Tt*CE15A–SeMet was screened for crystallization, and crystals grew in the same conditions as the native protein. A data set of *Tt*CE15A–SeMet was collected at beamline P11 of Petra III at 0.975 Å.

### Data processing and structure determination

Diffraction data were processed with XDS (32) with good statistics to 2.1 Å (Table S3), significantly better than the previously collected native data set, and was solely pursued for structure determination. Structure solution was carried out in Phenix (33). AutoSol (33, 34) was used to find the selenium sites and generate the initial SAD map with three protein molecules in the asymmetric unit. The figure of merit values before and after density modification in AutoSol were 0.34 and 0.64, respectively. The structure was initially built with Phenix AutoBuild (35), rebuilt in Coot (36), and further refined with Phenix Refine (37) in alternating cycles. Ligand compounds were added in Coot. The model has >97% residues in the most favorable Ramachandran regions with <0.2% Ramachandran outliers (38). Table S3 lists the final model refinement statistics.

### Inhibition studies

The aromatic compounds benzyl alcohol (BnzAlc), SA, FA, and *p*CoA (Sigma-Aldrich) as well as the carbohydrate compounds arabinose, glucose, xylose, xylotriose, and XUXX_R_ (Megazyme) were added to GE reactions, and the effect on activity of *Tt*CE15A (WT and variants) was monitored using the K-URONIC kit. Reactions with 400 μm BnzGlcA (significantly below the *K_m_*) were supplemented with the putative inhibitor in concentrations between 0 and 12 mm for carbohydrate compounds and BnzAlc, 0 and 4 mm for *p*CoA, or 0 and 1.2 mm for SA and FA. FA and SA concentrations above 1.2 mm resulted in too high background absorbance levels at the wavelength used in the coupled assay. Inhibition with only GlcA could not be performed due to the assay setup. IC_50_ values were obtained, where possible, by plotting the activity *versus* the concentration of inhibitor and fitting Equation 1 with nonlinear regression using OriginPro 2017 software.

(Eq. 1) $$k_{\text{obs}}=\frac{k_{\max}}{1+\frac{\left[I\right]}{{\text{IC}}_{50}}}$$

## Author contributions

J. A. B., S. M., L. L. L., and J. L. conceptualization; J. A. B., S. M., J.-C. N. P., L. O., L. L. L., and J. L. data curation; J. A. B., S. M., J.-C. N. P., L. O., L. L. L., and J. L. formal analysis; J. A. B., S. M., J.-C. N. P., L. O., L. L. L., and J. L. validation; J. A. B., S. M., J.-C. N. P., L. L. L., and J. L. investigation; J. A. B., S. M., and J. L. visualization; J. A. B., S. M., J.-C. N. P., L. L. L., and J. L. methodology; J. A. B., S. M., and J. L. writing-original draft; J. A. B., S. M., L. O., L. L. L., and J. L. writing-review and editing; L. O., L. L. L., and J. L. resources; L. O., L. L. L., and J. L. supervision; L. O., L. L. L., and J. L. funding acquisition; L. L. L. and J. L. project administration.

## Supplementary Material

Supporting Information

## Footnotes

4 Please note that the JBC is not responsible for the long-term archiving and maintenance of this site or any other third party hosted site.

## Footnotes

3 The abbreviations used are:

GE: glucuronoyl esterase
CE15: carbohydrate esterase family 15
LC: lignin-carbohydrate
GlcA: d-glucuronic acid
GalA: d-galacturonic acid
BnzGlcA: benzyl glucuronoate
AllylGlcA: allyl glucuronoate
MeGlcA: methyl glucuronoate
MeGalA: methyl galacturonoate
*p*NP-Ac: 4-nitrophenyl acetate
FA: ferulic acid
SA: sinapic acid
*p*CoA: *p*-coumaric acid
XUXX_R_: 2^3^-(4-*O*-methyl-d-glucuronyl)-α-d-xylotetraitol
*Tt*: *T. turnerae*
*Ot*: *O. terrae*
*Su*: *S. usitatus*
*Tr*: *T. reesei*
*St*: *S. thermophile*
*Sl*: *S. linguale*
*Sc*: *S. commune*
SAD: single-wavelength anomalous diffraction
BnzAlc: benzyl alcohol
Reg: region.
