# Supplementary material for: Structure–function analyses reveal that a glucuronoyl esterase from Teredinibacter turnerae interacts with carbohydrates and aromatic compounds
Source: J Biol Chem. 2019 Feb 27;294(16):6635–44. doi: 10.1074/jbc.RA119.007831 (PMC6484129; doi:10.1074/jbc.RA119.007831)
Supplement: Supporting Information [file supp_294_16_6635__index.html]

Structure-function analyses reveal that a glucuronoyl esterase from Teredinibacter turnerae interacts with carbohydrates and aromatic compounds — Characterization of the glucuronoyl esterase TtCE15A — Structure–function analyses reveal that a glucuronoyl esterase from Teredinibacter turnerae interacts with carbohydrates and aromatic compounds — Characterization of the glucuronoyl esterase TtCE15A — Supporting Information 

# Structure–function analyses reveal that a glucuronoyl esterase from *Teredinibacter turnerae* interacts with carbohydrates and aromatic compounds

## Supporting Information

- Supporting Information (to be published online) - Revised supporting information
