# Supplementary material for: Structure–function analyses reveal that a glucuronoyl esterase from Teredinibacter turnerae interacts with carbohydrates and aromatic compounds
Source: J Biol Chem. 2019 Feb 27;294(16):6635–44. doi: 10.1074/jbc.RA119.007831 (PMC6484129; doi:10.1074/jbc.RA119.007831)
Supplement: Supporting Information [file supp_RA119.007831_143500_1_supp_289401_pn8m57.pdf]

SUPPORTING INFORMATION

Structure-function analyses reveal that a glucuronoyl esterase from *Teredinibacter turnerae* interacs with carbohydrates and aromatic compounds

Authors: Jenny Arnling Bååth, Scott Mazurkewich, Jens-Christian Navarro Poulsen, Lisbeth Olsson, Leila Lo Leggio and Johan Larsbrink

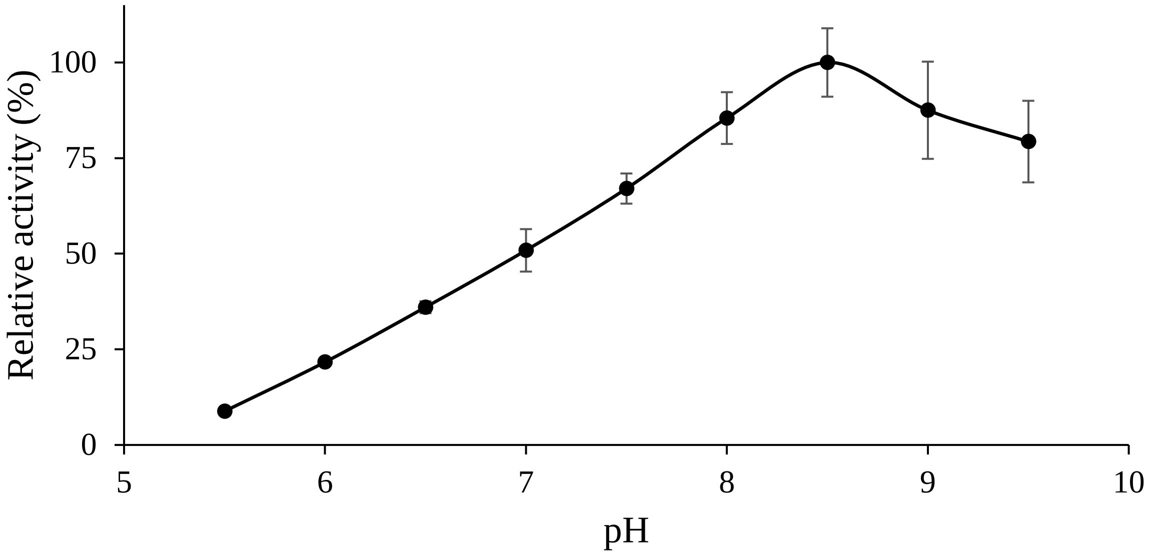

Figure S1. Effect of pH on hydrolysis of BnzGlcA by *Tt*CE15A. The pH-dependence was analyzed with 2 mM BnzGlcA as substrate in a three-component buffer containing 25 mM acetic acid, 25 mM MES, and 50 mM Tris-HCl, covering pH 4.5-9.5. Measurements at higher pHs were not possible due to substrate instability. Mean values are plotted with standard errors of the mean generated from duplicate measurements.

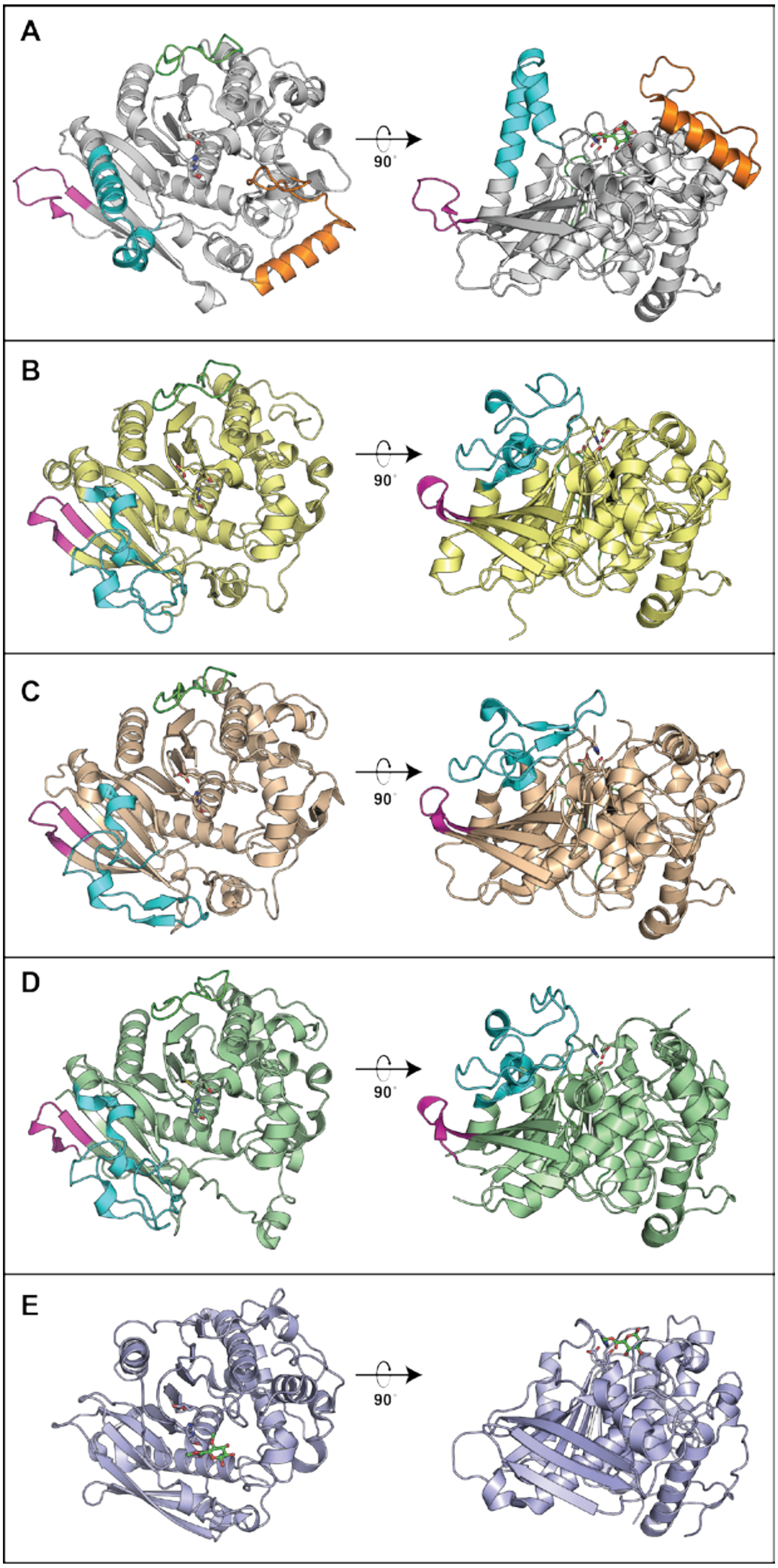

Figure S2. Comparison of structurally determined CE15 members. The overall structure of *Tt*CE15A (A, PDB: 6hsw), *Ot*CE15A from *Opitutus terrae* (B, PDB: 6gs0), *Su*CE15A from *Solibacter usitatus* (C, PDB: 6gry), MZ0003 from a marine metagenome analysis (D, PDB: 6ehn), and *St*GE2 from *Thermothelomyces thermophila* (E, PDB: 4g4j). The catalytic triad in each enzyme is shown as sticks and the methyl ester of 4-*O*-methyl glucuronoate is shown as green sticks with the *St*GE2 structure in panel E. The inserted regions in the bactertial enzymes, relative to their fungal counterparts, regions 1, 2, and 3, are colored magenta, cyan, and green, respectively. Region N only found in *Tt*CE15A is coloured orange.

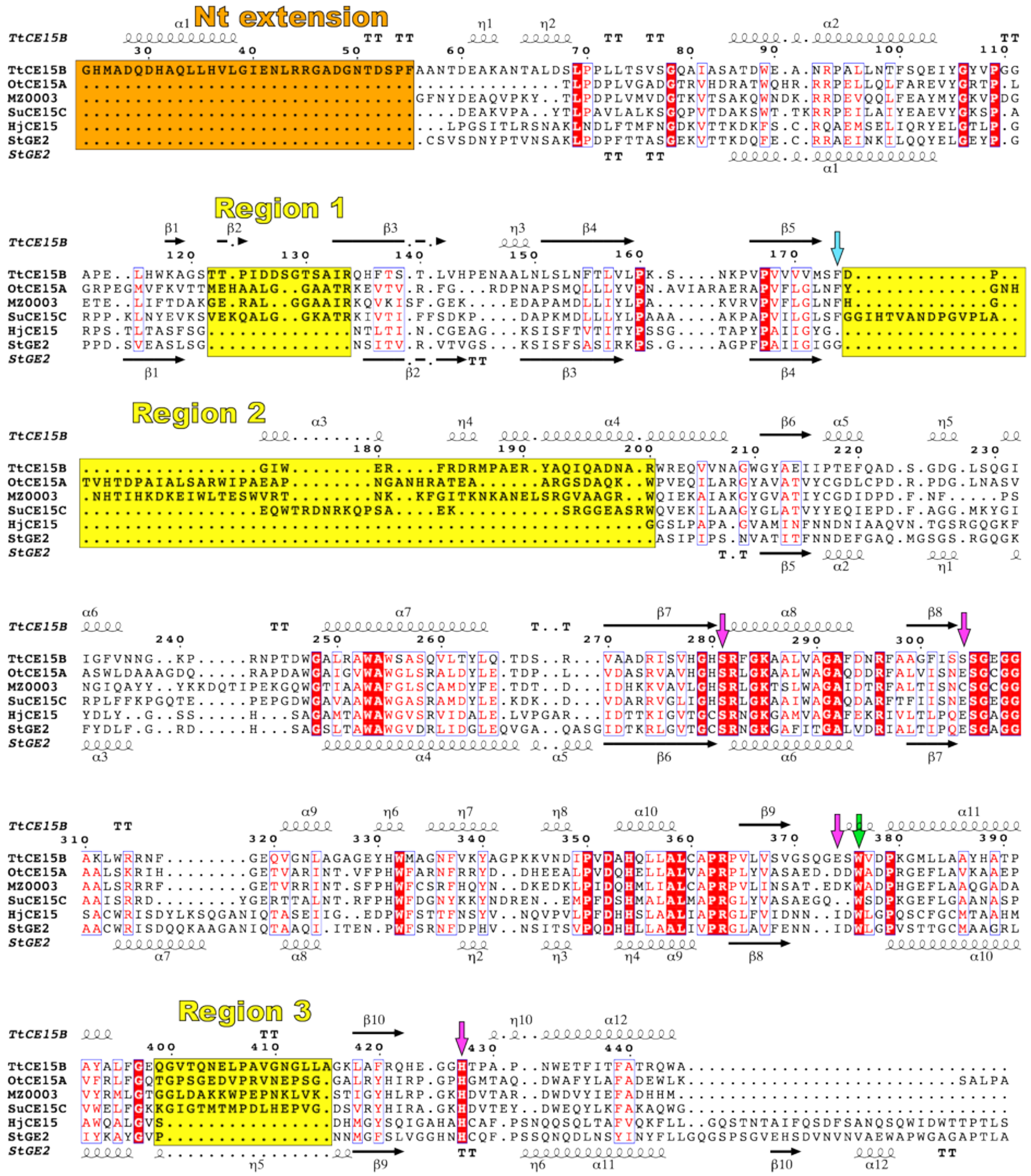

Figure S3. Structure-based sequence alignment of all structurally characterized glucuronoyl esterases. Similar residues are written in red text while conserved residues are written in white text over a red background. The inserted regions 1, 2 and 3 found in the bacterial structures relative to the fungal counterparts are highlighted in yellow. The N-terminal region (Nt extension; RegN) in *Tt*CE15A is highlighted in orange. The residues of the conserved catalytic triad, including the canonical and non-canonical acidic residues, are indicated with magenta arrows. The suggested “lignin-binding” Phe is indicated with a cyan arrow and the proposed “carbohydrate-binding” Trp with a green arrow.

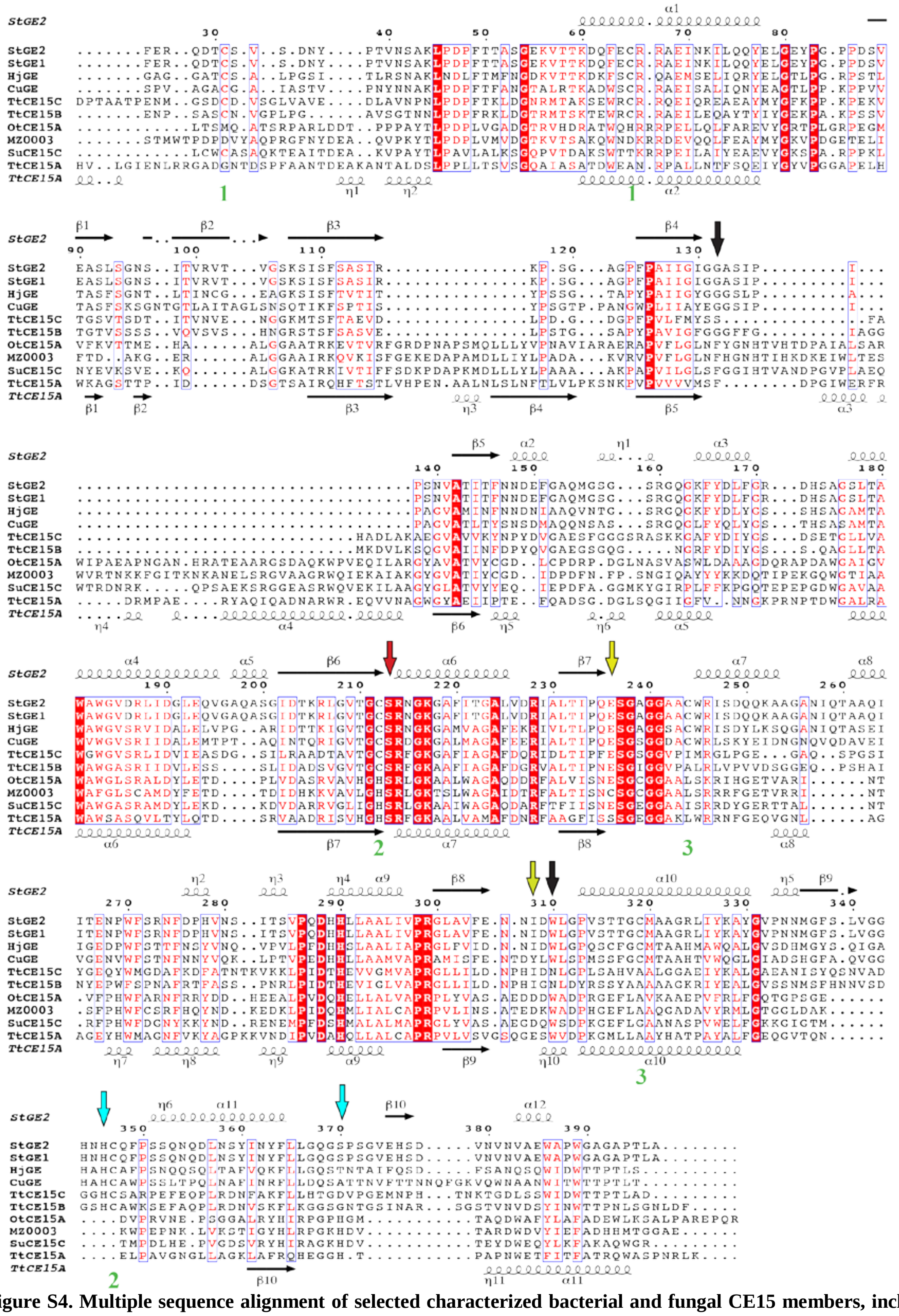

Figure S4. Multiple sequence alignment of selected characterized bacterial and fungal CE15 members, including the three CE15 enzymes encoded by *T. turnerae* (*Tt*CE15A, *Tt*CE15B and *Tt*CE15C). Similar residues are written in red text while conserved residues are written in white text over a red background. Arrows indicate significant residues in the active site. The conserved catalytic serine is indicated with a red arrow, histidine with a cyan arrow and the canonical or non-canonical glutamate/aspartate with a yellow arrow. Both *Tt*CE15B and *Tt*CE15C have the canonical glutamate and *Tt*CE15C additionally have an aspartate residue at the non-canonical position. Notably, the histidine of *Tt*CE15B and *Tt*CE15C align with the histidine of the fungal CE15 enzymes, whearase the histidine of the other bacterial enzymes align at a later point. The suggested "lignin and carbohydrate binding" phenylalanine and tryptophan residues are indicated with black arrows. Neither of these residues are conserved in *Tt*CE15B or *Tt*CE15C. Three disulfide bridges, conserved in the fungal GEs are labeled with green numbers. Two of these bridges are also conserved in *Tt*CE15B and *Tt*CE15C, but not in the other aligned bacterial sequences.

Table S1. Sequence identity (%) between *Tt*CE15A, the other CE15 enzymes encoded by *T. turnerae*, and previously characterized CE15 enzymes. Sequence identity (%) and query coverage (%, in brackets) were calculated using BLAST (https://blast.ncbi.nlm.nih.gov/Blast.cgi) with *Tt*CE15A as query sequence. *Tt*CE15A was aligned against *Tt*CE15B and C, all characterized bacterial CE15 enzymes, as well as the two structurally determined fungal CE15 enzymes.

**Origin**	**Enzyme**	**% Identity (% query coverage)**
T. turnerae	*Tt*CE15B	37 (57)
	*Tt*CE15C	25 (71)
Bacterial	*Ot*CE15A*	34 (88)
	*Ot*CE15B	32 (35)
	*Ot*CE15C	34 (96)
	*Ot*CE15D	41 (95)
	*Sl*CE15A	34 (94)
	*Sl*CE15B	39 (92)
	*Sl*CE15C	41 (99)
	*Su*CE15A	34 (91)
	*Su*CE15B	43 (99)
	*Su*CE15C*	33 (92)
	MZ0003*	30 (92)
Fungal	Cip2*	34 (37)
	*St*GE2*	32 (28)

*Structurally determined CE15 enzymes

Table S2. Primers used for cloning and mutagenesis of *Tt*CE15A.

Construct	Primer	5'-3' sequence
WT	TtCE15Af	ACTTCCAGGGCCATATGGCAGATCAGGATCACGCACAGCTACTTCAC
	TtCE15Ar	GGTGGTGGTGCTCGAGTTATGCCCATTGGCGCGTTGCAAAGGTGATAAA
S281A	TtA-S281Af	CCACGCACGCTTTGGCAAGGCGGCACTGG
	TtA-S281Ar	GCCAAAGCGTGCGTGGCCGTGGACACTGA
F174A	TtA-F174Af	ATGAGCGCCGACCCTGGCATTTGGGAGCGT
	TtA-F174Ar	GCCAGGGTCGGCGCTCATCACTACTACCACCGGT
F174D	TtA-F174Df	ATGAGCGATGACCCTGGCATTTGGGAGCGTTTC
	TtA-F174Dr	CAGGGTCATCGCTCATCACTACTACCACCGGTACG
W376A	TtA-W376Af	AGAGTGCAGTCGATCCGAAAGGTATGCTG
	TtA-W376Ar	ATCGACTGCACTCTCGCCCTGGCTGCC
W376D	TtA-W376Df	GAGAGTGACGTCGATCCGAAAGGTATGCT
	TtA-W376Dr	ATCGACGTCACTCTCGCCCTGGCTGC
E374A	TtA-E374Af	GGGCGCGAGTTGGGTCGATCCGAAAGGTATGCTGC
	TtA-E374Ar	AACTCGCGCCCTGGCTGCCGACGCTCA
S304E	TtA-S304Ef	CAGCGAGTCCGGCGAAGGCGGTGCAAAG
	TtA-S304Er	CCGGACTCGCTGATAAATCCCGCGGCGAATCG

Table S3. Table of crystallographic statistics.

	*Tt*CE15A-SeMet
Data Collection	
Date	July 26, 2017
Source	P11 at Petra III
Wavelength (Å)	0.975
Space group	$P3_121$
Cell dimensions	
a, *b*, *c* (Å)	121.17, 121.17, 198.20
α, β, γ (°)	90, 90, 120
No. of measured reflections	1868254 (177660)
No. of independent reflections	92246 (9051)
Resolution (Å)	2.147
R_{merge} (%)	5.987 (52.44)
$CC_{1/2}$	0.998 (0.609)
Mean I/σI	12.49 (1.32)
Completeness	99.79 (98.96)
Redundancy	20.3 (19.6)
Refinement	
R_{work}/R_{free}	0.164/0.208
No. atoms	
Protein	9703
Ligand/ions	199
Water	821
B-factors	
Protein	45.51
Ligand/ions	72.98
Water	46.16
RMSD	
Bond length (Å)	0.011
Bond angles (°)	1.05
PDB accession	6hsw
